# VEGF-B Levels in the Vitreous of Diabetic and Non-Diabetic Patients with Ocular Diseases and Its Correlation with Structural Parameters

**DOI:** 10.3390/medsci5030017

**Published:** 2017-08-09

**Authors:** Joana Mesquita, João Paulo Castro de Sousa, Sara Vaz-Pereira, Arminda Neves, Paulo Tavares-Ratado, Fátima M. Santos, Luís A. Passarinha, Cândida T. Tomaz

**Affiliations:** 1 CICS-UBI-Centro de Investigação em Ciências da Saúde, Universidade da Beira Interior, 6201-506 Covilhã, Portugal; joanamesquita3@gmail.com (J.M.); ftxsantos@gmail.com (F.M.S.); lpassarinha@fcsaude.ubi.pt (L.A.P.); 2 Faculty of Medical Sciences, Universidade da Beira Interior, 6201-506 Covilhã, Portugal; jpcastrosousa@netcabo.pt (J.P.C.-S.); ptavares@fcsaude.ubi.pt (P.T.-R.); 3 Department of Ophthalmology, Centro Hospitalar de Leiria, 2410-197 Leiria, Portugal; armindaneves@hotmail.com; 4 Department of Ophthalmology, Hospital de Santa Maria, 1649 – 035 Lisbon, Portugal; saravazpereira@gmail.com; 5 Department of Ophthalmology, Faculty of Medicine, Universidade de Lisboa, 1649 – 035 Lisbon, Portugal

**Keywords:** angiogenesis, diabetic retinopathy, enzyme-linked immunosorbent assay, vascular endothelial growth factor-B, VEGF inhibition, vitreous humor

## Abstract

Vascular endothelial growth factor B (VEGF-B) is one of the enigmatic members of the VEGF family. The knowledge gap about VEGF-B expression and how its levels are altered in diabetic eyes were the focus of this investigation that was addressed by comparing and correlating vitreous VEGF-B between diabetic and non-diabetic patients. VEGF-B levels were measured by enzyme-linked immunosorbent assay in vitreous samples (*n* = 33) from diabetic (*n* = 25) and non-diabetic (*n* = 8) patients. Results were compared between groups. Optical coherence tomography from diabetic patients was evaluated for central retinal thickness (CRT) and macular volume (MV). Mean vitreous VEGF-B concentration was higher in diabetic (18.82 ± 1.44 pg/mL) vs. non-diabetic patients (17.90 ± 0.32 pg/mL) (*p* = 0.006), and in proliferative diabetic retinopathy (PDR) (19.03 ± 1.52 pg/mL) vs. non-PDR (NPDR) patients (18.18 ±0.96 pg/mL) (*p* = 0.025). In diabetic retinopathy (DR) patients, correlation between VEGF-B and CRT (μm) was positive and moderate: *r_s_* = 0.441 (*p* ≤ 0.05) and the correlation between VEGF-B and MV (mm^3^) was positive and robust: *r_s_* = 0.716 (*p* ≤ 0.01). VEGF-B levels are overexpressed in vitreous of diabetic patients, and the levels are higher in developed stages of DR. Correlation results show that CRT and MV increase with increased levels of VEGF-B. Targeting VEGF-B inhibition may have therapeutic beneficial implications.

## 1. Introduction

Vascular endothelial growth factor A (VEGF-A) is the most studied member of the VEGF family and is an important regulator of angiogenesis and vascular permeability in physiological and pathological conditions. It induces proliferation and migration of endothelial cells and vascular permeability, resulting in angiogenesis in vivo [1]. Aiello [2] demonstrated that the levels of VEGF-A levels are increased in ocular fluids in patients with active retinal neovascularization associated with several ocular diseases in diabetic patients. Since it promotes angiogenesis in pathological conditions [3,4,5] VEGF-A is considered an important therapeutic target and a focus of considerable ophthalmologic research. 

Besides VEGF-A, there are other members of this family that may be of relevance for ocular disease. In particular, vascular endothelial growth factor B (VEGF-B) has been described in cardiovascular disease, diabetes, and cancer; however, its function in ophthalmology remains poorly understood, as it is the most controversial molecule of the VEGF family [3,6,7]. VEGF-B has been investigated in different tumor types, and its expression levels were found to be upregulated in cancer tissue [6]. Yang et al. studied the role of VEGF-B in promoting cancer metastasis and tumor invasion, referring to this molecule as a vascular remodeling factor and a target for cancer treatment [8].

VEGF-B was discovered in 1996 as a VEGF homolog [9,10]. It is expressed in two different isoforms: a heparin binding VEGF-B_167_ and diffusible VEGF-B_186_ [11], as seen in Table 1 [6,7,12,13,14]. VEGF-B binds selectively to vascular endothelial growth factor receptor-1 (VEGFR-1) [9] and neuropilin-1 (NRP-1), exerting its effects through binding to those receptors. VEGF-A, VEGF-B, and PIGF (placental growth factor) bind to VEGFR-1 via D2 domains while VEGF-A binds to VEGFR-2 through the domains D2 and D3 [15]. Platania and colleagues studied the interactions of VEGF antagonists/VEGF-A complexes in silico and showed the differences in the interactions and affinities, although higher affinities alone do not always translate to better efficacy [16]. 

Furthermore, Lyer [17] studied the structure of VEGF-B(10–108)•VEGFR-1D complex and demonstrated that the domain 2 of VEGFR-1 was the minimal binding domain needed for VEGF-B interaction. It was also showed that the topology of the studied dimer did not alter when bound to the receptor. The fact that VEGF-B interacts with VEGFR-1 but not with VEGFR-2 is explained by electrostatic surface potentials: VEGF-B has a negative charge showing an affinity for the basic interface of VEGFR-1 and no affinity for VEGFR-2 [17]. In addition, VEGF-B may form heterodimers with VEGF-A and PIGF, regulating the bioavailability and the activity of those growth factors to bind VEGFR-1 and NRP-1 [17]. VEGF-B is not stimulated by hypoxia, cytokines, hormones, or oncogenes [18]. 

VEGF-B was studied mainly in animal models; however, the expression of VEGF-B in human eyes and the changes in its levels in certain eye diseases, such as diabetic retinopathy (DR), need to be elucidated. A better understanding of the molecular pathological events triggered by the increase in VEGF expression, and specifically in VEGF-B, may be of fundamental importance for the development of new treatments for these pathologies. This is because the anti-angiogenic therapy is particularly promising for the treatment of neovascular diseases. Although the anti-angiogenic properties of VEGF-B are poor, VEGF-B promotes cell survival through its binding to VEGFR-1 and NRP-1, playing an important role as an anti-apoptotic molecule. It also suppresses growth through VEGFR-1 when it acts as a decoy for VEGF-A [12,19].

This project aims to provide a better understanding of the VEGF-B expression in human vitreous humor and its relationship with DR. It may contribute to the development of improved targeted anti-VEGF therapies for VEGF-driven eye diseases, with a lower occurrence of serious adverse events and ultimately for better effective therapeutic interventions. Thus, the main objective of this study was the quantification and comparison of VEGF-B levels in vitreous samples of diabetic and non-diabetic patients. Additionally, VEGF-B levels were compared between patients with non-proliferative diabetic retinopathy (NPDR) and proliferative diabetic retinopathy (PDR) and correlated with central retinal thickness (CRT) and macular volume (MV). In addition, the glucose levels in the vitreous were measured and compared between the study groups: diabetic versus non-diabetic patients and NPDR versus PDR.

## 2. Materials and Methods 

### 2.1. Participants

The study enrolled 33 patients: 25 diabetic patients with PDR (*n* = 19) or NPDR (*n* = 6) (13 women and 12 men) and a control group of 8 non-diabetic patients (2 women and 6 men) with rhegmatogenous retinal detachment (RRD). To the best of our knowledge, there are no reports on the measurement of the VEGF-B levels in vitreous humor of patients with ocular disease, making it difficult to use it as a reference value. Rhegmatogenous retinal detachment patients with no reports of other eye diseases or disorders were selected to serve as a control sample, minimizing the bias caused in the interpretation of the results. Only one eye from each patient was studied. 

### 2.2. Collection of Samples and Data from Patients

Undiluted vitreous humor samples were collected from patients who were referred to the hospital for pars plana vitrectomy (PPV). 

These samples were collected at the beginning of the PPV (core vitrectomy). Tubes from the vitrectomy were disconnected and connected to a syringe (in coordination with vitrectomy aspiration at the beginning of the surgery). Before turning on the intravitreal infusion, an undiluted sample of vitreous was obtained by aspiration into a 2 mL syringe attached to the vitreous cutter. The sample was transferred to sterilized tubes, placed immediately on ice, and frozen at −80 °C until further analysis. 

Approximately 500 samples were collected from vitrectomized patients with several pathologies; of those, 33 samples were chosen for this study based on the following criteria: (1) patients with a confirmed diagnostic of DR (for the diabetic patient’s study arm), either with PDR or NPDR; (2) patients naïve to aflibercept treatment; and (3) patients with a sample volume that would allow the confirmation of the results through repeated enzyme-linked immunosorbent assay (ELISA) tests (Wuhan, China) for the determination of VEGF-B levels.

Quantitative analysis of the optical coherence tomography (OCT) scans was performed to evaluate CRT (μm) and MV (mm^3^) from DR patients using SPECTRALIS OCT (Heidelberg Engineering, Heidelberg, Germany).

Moreover, patient clinical history was investigated to confirm patient diagnosis, baseline characteristics, and associated concomitant diseases. Information about concomitant medications or non-therapeutic drugs that may influence the results was collected, including the use of previous anti-angiogenic drugs.

### 2.3. Measurement of Vitreous Vascular Endothelial Growth Factor B Levels

Quantification of vitreous VEGF-B was performed using ELISA Kit for VEGF-B for human samples (product SEA144Hu, USCN Life Sciences, Wuhan, China). Detection range was between 15.63–1000 pg/mL, and the sensitivity or the minimum detectable level was less than 5.5 pg/mL. The assay was conducted according to the protocol specified by the kit manufacturer. 

### 2.4. Measurement of Vitreous Glucose Levels 

The enzymatic UV test (hexokinase method) was used for specific quantitative determination of glucose in vitreous using a Beckman Coulter analyzer AU2700 (Brea, CA, USA) and Glucose reagent OSR6521. The results were reported in mmol/L.

### 2.5. Statistical Analysis

Statistical analysis was performed with SPSS (Statistical Package for Social Sciences) version 20.0 for Windows (Microsoft, Armonk, NY, United States of America). Student’s *t*-test and Mann–Whitney tests were used to analyze differences. Values of vitreous VEGF-B and glucose levels are reported as mean ± standard deviation (pg/mL or mmol/L, respectively). For all statistical analyses, a *p*-value ≤ 0.05 was considered to indicate statistically significant differences. Spearman’s ordinal correlation coefficient was used to analyze the relationship between quantitative variables because the variables had no normal distribution.

### 2.6. Ethical Statement

The study was approved by the Comissão de Ética para a Saúde (CES)/Ethics Committee for Health of the Centro Hospitalar de Leiria (Code: CHL-15481). All patients included in this study, which adhered to the tenets of the Declaration of Helsinki, gave their informed consent.

## 3. Results

### 3.1. Study Population—Baseline Characteristics

The baseline characteristics of both groups are shown in Table 2. The mean age of the diabetic and control groups was 68.21 ± 9.59 years and 61.50 ± 14.87 years, respectively. Six patients (24%) had NPDR, and the other 19 diabetic patients (76%) had PDR. Of the 25 patients in the diabetic group, 19 had prior laser treatment; of which 2 patients received a prior intravitreal injection of triamcinolone acetonide and 1 received previous treatment with anti-VEGF therapy, namely ranibizumab. None of the diabetic patients included in the study had been previously treated with aflibercept.

### 3.2. Comparison of Vascular Endothelial Growth Factor B Levels in Vitreous Humor between Diabetic and Control Groups

**Figure 1:**
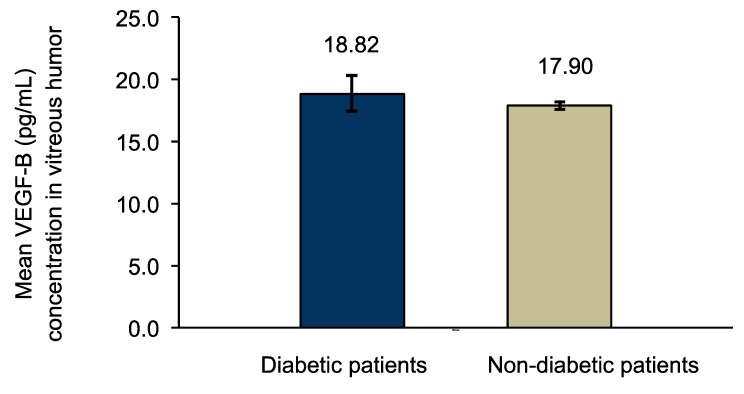
Comparison of VEGF-B vitreous levels between diabetic patients (*n* = 25) and non-diabetic patients (*n* = 8), analyzed with independent samples, *t*-test (*p* = 0.006).

The mean VEGF-B concentration in vitreous humor was higher in the diabetic patients’ group (18.82 ± 1.44 pg/mL) in comparison with the control group of non-diabetic patients (17.90 ± 0.32 pg/mL) (Figure 1). This difference was considered statistically significant (*p* = 0.006).

### 3.3. Comparison of Vascular Endothelial Growth Factor B Levels in Vitreous Humor between Patients with Proliferative Diabetic Retinopathy vs. Non-Proliferative Diabetic Retinopathy

**Figure 2:**
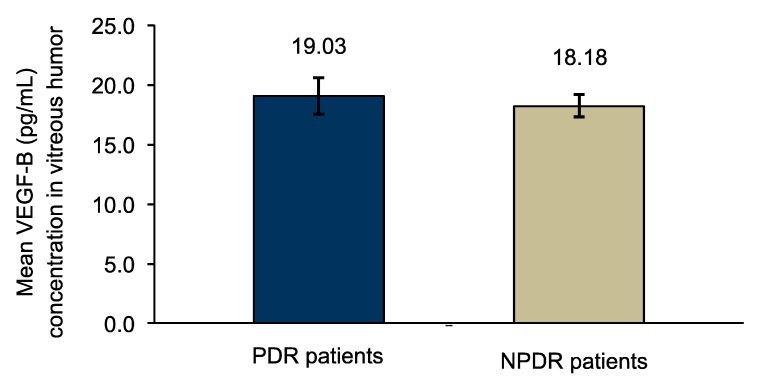
Comparison of VEGF-B levels in vitreous between PDR (*n* = 19) and NPDR patients (*n* = 6), analyzed with Mann–Whitney test (*p* = 0.025).

Mean VEGF-B measurements were higher in vitreous humor of patients with PDR (19.03 ± 1.52 pg/mL) vs. NPDR (18.18 ± 0.96 pg/mL), and this difference was statistically significant (*p* = 0.025) (Figure 2). 

### 3.4. Comparison of Glucose Vitreous Humor Levels between Patients with Diabetic Disease and the Control Group

**Figure 3:**
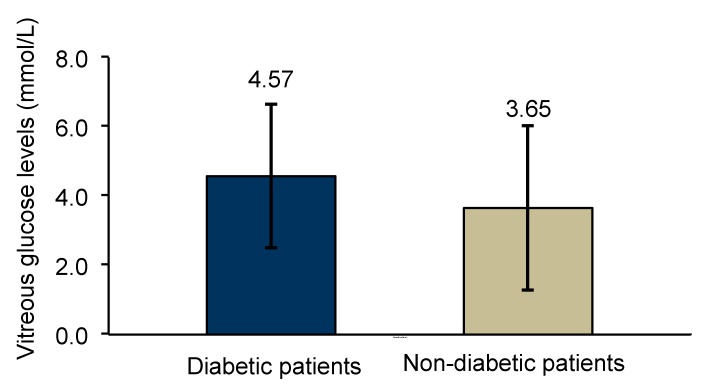
Comparison of glucose vitreous levels between diabetic (*n* = 25) and non-diabetic control group (*n* = 8) analyzed with independent-samples, *t*-test (*p* = 0.300).

Diabetic patients showed increased levels of glucose in vitreous humor (4.57 ± 2.07 mmol/L) when compared with non-diabetic patients (3.65 ± 2.39 mmol/L), but this difference was not statistically significant (*p* = 0.300) (Figure 3).

### 3.5. Comparison of Glucose Vitreous Humor Levels Between Patients with Proliferative Diabetic Retinopathy vs. Non-Proliferative Diabetic Retinopathy

**Figure 4:**
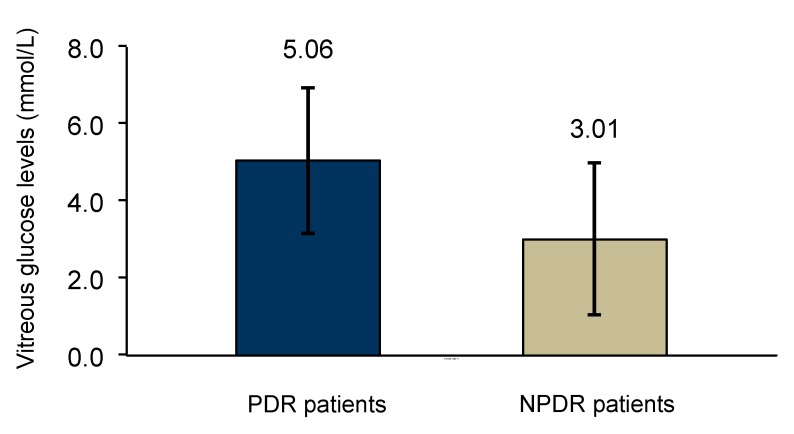
Comparison of glucose vitreous levels between NPDR (*n* = 6) and PDR patients (*n* = 19), analyzed with independent-samples, *t*-test (*p* = 0.032).

In this analysis, patients with PDR showed to have statistically higher values of glucose in vitreous when compared to NPDR group of patients (5.06 ± 1.89 mmol/L vs. 3.01 ± 1.98 mmol/L; *p* = 0.032) (Figure 4). 

### 3.6. Correlation between Vitreous Vascular Endothelial Growth Factor B in Diabetic Retinopathy Patients and Quantitative Measurements by Optical Coherence Tomography

**Figure 5:**
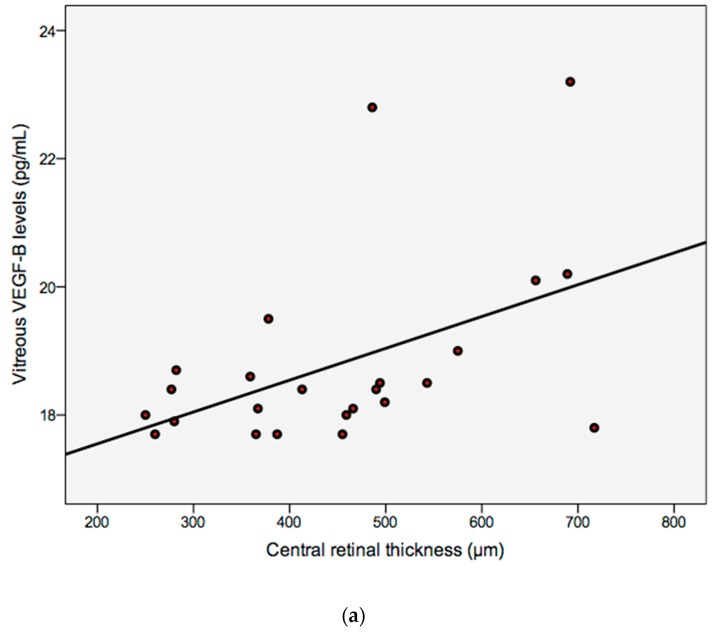

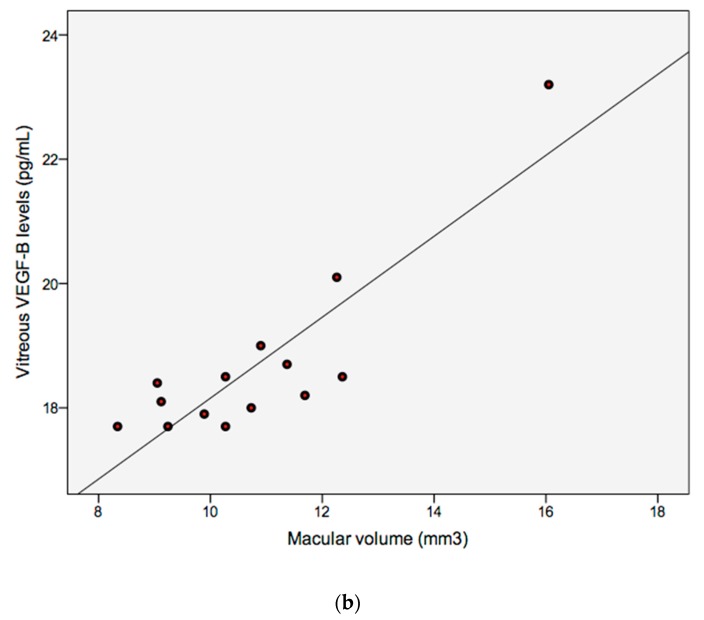
Correlation between vitreous VEGF-B (pg/mL) levels in DR patients (**a**) and central retinal thickness (CRT; μm), *r_s_* = 0.441 (*p* ≤ 0.05); or (**b**) and macular volume (MV; mm^3^), *r_s_* = 0.716 (*p* ≤ 0.01), analyzed using Spearman’s correlation coefficient.

The correlation between vitreous VEGF-B of DR patients and CRT was statistically significant, positive, and moderate: *r_s_* = 0.441 (*p* ≤ 0.05), as seen in Figure 5a. Moreover, the correlation between vitreous VEGF-B and MV in these patients was statistically significant, positive, and robust: *r_s_* = 0.716 (*p* ≤ 0.01), as observed in Figure 5b.

## 4. Discussion

VEGF-B is a proangiogenic cytokine [20] that has been implicated in several diseases. Contrary to VEGF-A, the prototypical member of the family that was found increased in the vitreous humor of patients with retinal diseases such as DR, the biological role of VEGF-B in the eye has not been sufficiently studied. Moreover, its function remains controversial and needs to be elucidated, specifically in pathological events in eye disease.

The present study demonstrated that VEGF-B is significantly increased in diabetic patients with ocular disease in comparison with non-diabetic patients. This is an interesting finding as the VEGF-B levels have not been measured in the vitreous humor of human eyes. Moreover, the levels of VEGF-B were found increased in PDR in comparison to NPDR, and this difference was statistically significant. In addition, we found a positive correlation between VEGF-B vitreous levels and the CRT or MV measured by OCT, suggesting that overexpression of VEGF-B may influence these quantitative parameters in DR patients.

It is uncertain whether the inhibition of VEGF-B is beneficial and improves patients’ visual acuity as well as structural outcomes, given the protecting and survival action of VEGF-B.

Reports on the role of VEGF-B have provided controversial findings. Silvestre [21] demonstrated that VEGF-B in mice had angiogenic properties, in part through VEGFR-1. Also, Wafai et al. [22] showed that VEGF-B under certain conditions promoted angiogenesis in the tibidis anterior muscle of rabbits with bilateral hind limb ischemia. Aase et al. [23] revealed that VEGF-B was necessary for heart function but not required for heart development or the angiogenesis process in the adult mice. Reichelt et al. [24] demonstrated that under normal conditions, the retinal vasculature of the mice was not affected by the lack of VEGF-B. Rissanen [25] also showed, with his experience with skeletal muscles of rabbits, that VEGFR-1 ligand VEGF-B did not induce angiogenesis. Bhardwaj et al. [26] studied the angiogenic responses of the different members of the VEGF family in rabbit carotid arteries and verified that VEGF-B did not display angiogenic activity. Li et al. [27] suggested a specific role of VEGF-B in the revascularization of ischemic myocardium, playing an important role as cardioprotector. In this specific case, VEGF-B was described as having angiogenic activity. In the same light, Mould [28] proposed VEGF-B therapy for ischemic heart disease due to its role in promoting vascular growth and, therefore, revascularization. An interesting finding by Li et al. [14] was that VEGF-B was a potent inhibitor of apoptosis, rescuing neurons in the retina from apoptosis in a mice model in ocular neurodegenerative diseases and the brain of a mice model of stroke. They also showed that the treatment with VEGF-B using an effective dose for the survival of neurons did not cause angiogenic activity. 

VEGF-B therapy may lead to vessel survival and rescue of blood vessels from apoptosis; thus, it is being referred as a possible therapy in neurologic diseases, such as Parkinson’s disease. Confirming these findings, Poesen et al. [29] showed the function of VEGF-B_186_ in the nervous system in an animal study. Specifically, the neuroprotective effects of VEGF-B in combination with its very low angiogenic and permeability action. They reported its role as a neuroprotector, turning it into an appealing treatment for neurodegenerative diseases. Based on the findings of the above studies, it can be stated that VEGF-B plays a role in response to pathological conditions, and its upregulation may demonstrate benefits.

However, other studies reported that VEGF-B plays a role as an inhibitor of choroidal and retinal neovascularization through VEGFR-1 and NRP-1. In his work, Zhang et al. [30] referred to VEGF-B as a survival factor instead of an angiogenic factor, suggesting that the survival factor was mediated by NRP-1 and VEGFR-1 complexes. Li et al. [19] reported that VEGF-B targeting inhibits pathological angiogenesis by abolishing blood cell survival, confirming the apoptotic effect of VEGF-B. Li et al. [12] also referred that antigrowth and anti-angiogenic effects of the VEGF-B are mediated through VEGFR-1, which acts as a VEGF-A decoy, suppressing angiogenesis. 

Based on the above studies describing the different functions of VEGF-B and the results of our investigation where VEGF-B appears to be overexpressed in DR in comparison with RRD, we suggest that VEGF-B targeted therapy by an anti-angiogenic drug may have a therapeutic effect on neovascular diseases. Due to the inhibition of VEGF-B, the treatment of vascular diseases of the retina may improve outcomes and have positive effects on the patients. It is important to acknowledge that VEGF-B does not act under normal physiological conditions, but instead in pathological conditions, which makes VEGF-B an attractive and safe therapeutic molecule.

Lundquist O. and Österlin [31] demonstrated in their study that vitreous glucose concentration was in general (with some exceptions) lower than in blood but was higher in the diabetic group than in the non-diabetic group of patients. In our study, glucose concentration was also assayed in vitreous humor samples obtained from patients of the two study groups. The diabetic patients showed higher glucose levels in the vitreous than the non-diabetic group; however, the difference was not statistically significant. Moreover, vitreous glucose concentration was lower in NPDR patients than in the PDR patients, suggesting that vitreous glucose concentration increases with disease severity.

## 5. Conclusions

VEGF-B was found significantly increased in DR patients, and this increase is significantly higher as the DR is at a more advanced stage. We suggest VEGF-B can offer an alternative and challenging therapeutic target in the treatment of neovascular conditions, such as diabetic eye disease. Randomized clinical trials are needed to be conducted in order to confirm the efficacy of anti-VEGF-B therapy. Furthermore, due to the cardioprotective and neuroprotective effect of this molecule, and taking into account that diabetic patients have multiple disease risk factors, studies targeting VEGF-B inhibition must be conducted carefully to determine its safety, particularly in these patients.

## Figures and Tables

**Table 1:** Summary of the characteristics of Vascular endothelial growth factor B (VEGF-B).

Characteristics	
Exons	7
2 Isoforms	VEGF-B_167_—heparin binding isoform, carboxyl terminus
	VEGF-B_186_—diffusible isoform, hydrophobic carboxyl terminus
Binding	VEGFR-1 and NRP-1
Molecular masses of homodimers	VEGF-B_167_ = 42 kDa
	VEGF-B_186_ = 60 kDa
Expression	In various tissues, as the heart and skeletal muscle, vascular smooth muscles, endothelium cells, mural cells, pericytes, smooth muscle cells, vascular stem/progenitor cells. Overall, it is expressed in tissues with high metabolic activity such as the myocardium.
Role	VEGF-B was described as playing roles in angiogenesis, however its ability to stimulate directly angiogenesis is poor. VEGF-B has an important role in the heart (cardioprotection), neurologic diseases (inducing neuroprotection), cancer and metabolic diseases.

VEGFR:-1: vascular endothelial growth factor receptor 1 ; NRP-1: neuropilin-1 . [6,7,12,13,14].

**Table 2:** Patient baseline clinic, demographic characteristics, and previous treatments

Patient Number	Gender	Eye Submitted to PPV	Diagnosis and/or Stage of Severity of Diabetic Retinopathy	Previous Treatment for Diabetic Retinopathy (before Vitrectomy)
1	F	OD	NPDR	No treatment
2	M	OS	PDR	Laser therapy
3	M	OS	PDR	Laser therapy
4	M	OD	NPDR	No treatment
5	M	OD	PDR	Laser therapy plus intravitreal triamcinolone acetonide
6	F	OS	PDR	Laser therapy
7	F	OD	PDR	Laser therapy
8	M	OD	NPDR	No treatment
9	F	OS	PDR	Laser therapy plus ranibizumab intravitreal injection
10	F	OD	PDR	Laser therapy
11	F	OS	NPDR	No treatment
12	M	OS	PDR	Laser therapy
13	F	OD	PDR	Laser therapy
14	M	OS	PDR	Laser therapy
15	M	OS	NPDR	Laser therapy
16	F	OD	PDR	Laser therapy
17	M	OD	PDR	Laser therapy
18	F	OD	PDR	Laser therapy
19	M	OD	PDR	Laser therapy plus intravitreal triamcinolone acetonide
20	F	OD	PDR	Laser therapy
21	F	OD	NPDR	No treatment
22	M	OD	PDR	Laser therapy
23	F	OD	PDR	Laser therapy
24	F	OD	PDR	Laser therapy
25	M	OS	PDR	Laser therapy
26	M	OD	RRD	Not applicable
27	M	OS	RRD	Not applicable
28	M	OS	RRD	Not applicable
29	M	OS	RRD	Not applicable
30	M	OD	RRD	Not applicable
31	M	OS	RRD	Not applicable
32	F	OS	RRD	Not applicable
33	F	OD	RRD	Not applicable

F: Female; M: Male; PPV: Pars plana vitrectomy; OD: right eye; OS: left eye; PDR: Proliferative diabetic retinopathy; NPDR: Non-proliferative diabetic retinopathy; RRD: Rhegmatogenous retinal detachment.
